# Supplementary material for: Development and Characterization of Cationic Nanostructured Lipid Carriers as Drug Delivery Systems for miRNA-27a
Source: Pharmaceuticals (Basel). 2023 Jul 14;16(7):1007. doi: 10.3390/ph16071007 (PMC10384247; doi:10.3390/ph16071007)
Supplement: Supplementary file 1 [file pharmaceuticals-16-01007-s001.zip › pharmaceuticals-2443573-supplementary.pdf]

pharmaceuticals MDPI

Supplementary material

Table S1. LD data of NLC formulations that contain a different concentration of octadecylamine (0–0.15%, w/w) in a function of time (30, 60, 90, 180 days, and 270 days stored at three different temperature conditions

Storage conditions	*Formulation*	*1 month*			*2 months*			*3 months*			*6 months*			*9 months*		
		$d_{(0.5)}$	$d_{(0.9)}$	$d_{(0.99)}$	$d_{(0.5)}$	$d_{(0.9)}$	$d_{(0.99)}$	$d_{(0.5)}$	$d_{(0.9)}$	$d_{(0.99)}$	$d_{(0.5)}$	$d_{(0.9)}$	$d_{(0.99)}$	$d_{(0.5)}$	$d_{(0.9)}$	$d_{(0.99)}$
4 ± 3 °C	bNLC	0.128 ± 0.000	0.188 ± 0.002	0.240 ± 0.001	0.128 ± 0.000	0.189 ± 0.001	0.250 ± 0.000	0.129 ± 0.002	0.191 ± 0.002	0.250 ± 0.000	0.134 ± 0.002	0.198 ± 0.002	0.253 ± 0.006	0.130 ± 0.001	0.194 ± 0.001	0.250 ± 0.000
	cNLC-2	0.125 ± 0.001	0.205 ± 0.000	0.270 ± 0.000	0.126 ± 0.000	0.232 ± 0.002	0.331 ± 0.001	0.130 ± 0.000	0.191 ± 0.000	0.250 ± 0.000	0.130 ± 0.000	0.191 ± 0.000	0.250 ± 0.000	0.130 ± 0.000	0.193 ± 0.000	0.250 ± 0.000
	cNLC-3	0.129 ± 0.000	0.228 ± 0.000	0.270 ± 0.000	0.129 ± 0.000	0.191 ± 0.000	0.250 ± 0.000	0.130 ± 0.000	0.192 ± 0.000	0.230 ± 0.000	0.131 ± 0.000	0.192 ± 0.000	0.250 ± 0.000	0.130 ± 0.000	0.193 ± 0.000	0.250 ± 0.000
25 ± 2 °C (60 ± 5% RH)	bNLC	0.129 ± 0.000	0.227 ± 0.000	0.250 ± 0.000	0.128 ± 0.000	0.189 ± 0.001	0.220 ± 0.000	0.134 ± 0.002	0.196 ± 0.002	0.253 ± 0.006	0.136 ± 0.001	0.200 ± 0.001	0.260 ± 0.000	0.150 ± 0.005	0.265 ± 0.004	0.360 ± 0.000
	cNLC-2	0.121 ± 0.000	0.207 ± 0.000	0.240 ± 0.000	0.128 ± 0.000	0.188 ± 0.000	0.240 ± 0.001	0.129 ± 0.001	0.189 ± 0.000	0.240 ± 0.000	0.130 ± 0.000	0.191 ± 0.000	0.251 ± 0.000	0.129 ± 0.000	0.190 ± 0.000	0.250 ± 0.000
	cNLC-3	0.122 ± 0.000	0.226 ± 0.001	0.290 ± 0.001	0.129 ± 0.001	0.190 ± 0.000	0.250 ± 0.000	0.130 ± 0.001	0.190 ± 0.000	0.250 ± 0.000	0.130 ± 0.000	0.191 ± 0.000	0.250 ± 0.000	0.129 ± 0.000	0.191 ± 0.000	0.250 ± 0.000
40 ± 2 °C (75 ± 5% RH)	bNLC	0.132 ± 0.000	0.220 ± 0.002	0.230 ± 0.000	0.128 ± 0.001	0.170 ± 0.000	0.230 ± 0.000	0.130 ± 0.000	0.190 ± 0.001	0.240 ± 0.000	0.142 ± 0.002	0.230 ± 0.001	0.293 ± 0.006	0.141 ± 0.004	0.229 ± 0.009	1.223 ± 0.014
	cNLC-2	0.126 ± 0.001	0.208 ± 0.000	0.271 ± 0.000	0.128 ± 0.000	0.165 ± 0.002	0.220 ± 0.000	0.128 ± 0.001	0.187 ± 0.002	0.240 ± 0.000	0.139 ± 0.001	0.210 ± 0.004	0.270 ± 0.000	0.138 ± 0.001	0.222 ± 0.002	0.283 ± 0.006
	cNLC-3	0.122 ± 0.000	0.221 ± 0.002	0.250 ± 0.000	0.129 ± 0.000	0.161 ± 0.001	0.220 ± 0.000	0.128 ± 0.001	0.195 ± 0.001	0.240 ± 0.000	0.133 ± 0.000	0.195 ± 0.000	0.250 ± 0.000	0.129 ± 0.000	0.191 ± 0.000	0.250 ± 0.001
